# Efficiency of time-restricted eating and energy restriction on anthropometrics and body composition in adults: a systematic review and meta-analysis of randomized controlled trials

**DOI:** 10.1186/s12966-025-01812-w

**Published:** 2025-09-29

**Authors:** Yichao Sun, Yubo Liu, Weibing Ye, Veeranjaneya Reddy Lebaka, Venkatrayulu Chenji, Weiping Li, Mallikarjuna Korivi

**Affiliations:** 1 Institute of Human Movement and Sports Engineering, College of Physical Education and Health Sciences, Zhejiang Normal University, Jinhua, 321004 Zhejiang China; 2 Department of Microbiology, Yogi Vemana University, Kadapa, India; 3 Department of Marine Biology, Vikrama Simhapuri University, Nellore, 524324 India; 4 Heavy Athletic Training Center of Guangdong Province, Guangzhou, Guangdong China

**Keywords:** Body weight, Fat mass, Obesity, Fasting, Daily eating window, Dietary therapy

## Abstract

**Background:**

We examined the effect of time-restricted eating (TRE) alone and in combination with energy restriction (ER) on the body composition in adults.

**Methods:**

PubMed, the Cochrane Library, EMBASE, Web of Science, and Scopus were systematically searched for articles that evaluated the effects of TRE or TRE + ER on body composition variables. Meta-analysis was performed to explore the overall effect of TRE on body composition. Subgroup analysis was performed to compare the changes in the TRE and TRE + ER trials with those in no restriction (NR) and ER trials. Intervention effects on body composition are expressed as weighted mean differences (WMDs) and 95% confidence intervals (CIs).

**Results:**

A total of 20 randomized controlled trials (1242 participants) were included in the systematic review and meta-analysis. Pooled results showed that TRE significantly decreased body weight, fat mass, fat-free mass, body mass index (BMI) and waist circumference (WC). Subgroup analyses revealed that TRE alone (compared to NR) and TRE plus ER (compared to ER) considerably decreased body weight (TRE = WMD:-1.59 kg, 95%CI:-2.02 to -1.15 *p* < 0.00001; TRE + ER = WMD:-0.94 kg, 95%CI:-1.58 to -0.31, *p* = 0.004) and fat mass (TRE = WMD:-0.93 kg, 95%CI:-1.22 to -0.63, *p* < 0.00001; TRE + ER = WMD:-1.13 kg, 95%CI:-1.50 to -0.75, *p* < 0.00001) but not fat mass percentage. Both TRE and TRE + ER decreased fat-free mass (TRE = WMD:-0.58 kg, 95%CI:-0.81 to -0.36, *p* < 0.00001; TRE + ER = WMD:-0.56 kg, 95%CI:-0.81 to -0.31, *p* < 0.0001). TRE independently and combinedly decreased BMI (TRE = WMD:-0.63 kg/m^2^, 95%CI:-0.95 to -0.30, *p* = 0.0002; TRE + ER = WMD:-0.42 kg/m^2^, 95%CI:-0.78 to -0.07, *p* = 0.02) and WC (TRE = WMD:-2.11 cm, 95%CI:-3.35 to -0.86, *p* = 0.0009; TRE + ER = WMD:-2.20 cm, 95%CI: -2.64 to -1.75, *p* < 0.00001).

**Conclusions:**

TRE independently promotes weight loss in adults, and combining of TRE with ER has no additional beneficial effects on weight loss.

**Trial registration:**

This study was registered with PROSPERO: CRD42022370215.

**Supplementary Information:**

The online version contains supplementary material available at 10.1186/s12966-025-01812-w.

## Background

Excessive or improper food intake due to changes in dietary patterns or eating habits over a period of time is associated with weight gain and subsequent metabolic disorders [1]. Proper intake and expenditure of food, referred to as ‘energy balance’, is directly involved in the management of body weight, and ‘energy imbalance’ leads to change in body composition [2, 3]. Excessive intake of food without controlling energy or eating window in a day is said to be the primary contributor to overweight and obesity [4, 5]. Obesity is characterized by excessive adipose tissue and uneven body composition in adults, and is closely associated with the prevalence of type 2 diabetes, hypertension, fatty liver, and cardiovascular disease, and even early death [5–7]. Healthy eating behavior with a steady state of daily fasting:eating cycle is crucial for maintaining stable health, body composition, and physical fitness [8, 9]. Clinically, anthropometric and body composition indices can be assessed by measuring the changes in body weight, body mass index (BMI), waist circumference (WC), fat mass, and fat-free mass [10]. Previous studies have shown that controlling daily energy intake by energy/calorie restriction (CR) or restricting daily eating window through fasting are alternative approaches to prevent or manage obesity and metabolic disorders [11, 12].

Time-restricted eating (TRE) is a type of intermittent fasting (alternate to CR) that allows daily intake of food within a time window of 4 to 12 h [13–15]. TRE primarily controls the daily eating hours but not the intake of energy or composition of a meal [14]. Practicing TRE has gained popularity in recent years due to its beneficial effects on weight management and cardiometabolic health in people with overweight or obesity [16, 17]. TRE reported to attenuate diet-induced metabolic diseases, including diabetes, liver steatosis, and hypercholesterolemia [16–19]. Recent randomized controlled trials (RCTs) have shown that TRE with exercise intervention can decrease body weight and fat mass in adults with overweight and obesity [20, 21]. On the other hand, TRE often causes unintentional restriction of energy intake based on daily eating/fasting hours, which also results in weight loss, decreased blood pressure, and improved cardiometabolic fitness [17, 22–24]. The beneficial effects of TRE are said to be associated with the number of fasting hours per day or combination of other interventions [14, 18, 22–24]. In this context, it is unclear whether the beneficial effects of TRE are due to the unintentional energy restriction (ER), fasting hours, and/or a combination of both [25]. Some evidence suggests that TRE is not more beneficial than ER in the reduction of body weight, fat mass or metabolic risk factors in patients with obesity [12]. While other studies stated that ER alone can promote weight loss and reduce diabetes risk factors in adults with overweight and obesity [24, 26]. However, adherence to ER protocol (61%) is a challenge to participants [27], whereas practicing TRE is easier (adherence > 84%), in which participants do not need to reduce their daily food intake or counting calories [19, 27–29].

As the number of RCTs on TRE interventions are increasing, recent systematic reviews and meta-analyses have summarized the practical implications of TRE on various clinical outcomes in people with normal weight, overweight or obesity [30–32]. Specifically, these studies suggest TRE as a potential therapeutic strategy for managing anthropometric or body composition measures, including body weight, fat mass, and WC [19, 31–33]. However, the trials included in these meta-analyses were not exclusively TRE, instead they were combined with other interventions, like ER [34] or exercise [19, 31]. Furthermore, a recent meta-analysis has shown that TRE is less effective than ER or alternate-day fasting for weight loss, though the underlying reasons remain unexplored [30]. The inconsistent findings of these meta-analyses may stem from the differences in the TRE protocols and/or using of unsuitable comparator trials. Given these limitations, synthesizing evidence using RCTs is important to explore the independent (TRE) or combination (TRE + ER) effects of TRE on body composition. To date, no systematic review and meta-analysis has specifically addressed whether adding ER to TRE (without exercise) enhances the beneficial effects than TRE alone on the anthropometric and body composition variables. Since meta-analysis provides comprehensive evidence in medical research [35], we proposed this systematic review and meta-analysis to evaluate the independent effect of TRE, as well as the combined effects of TRE plus ER on body composition in adults. We hypothesized that TRE combined with ER may have additional beneficial effects than that of TRE alone-induced beneficial effects on body composition.

## Methods

### Literature search strategy

Literature search was conducted according to the latest guidelines of the Preferred Reporting Items for Systematic Reviews and Meta-Analyses (PRISMA) [36]. The protocol of this systematic review and meta-analysis was registered in the International Prospective Register of Systematic Reviews (PROSPERO) with the ID CRD42022370215. PRISMA checklist with the details of items in our study was provided as a Supplementary Table 1.

Major electronic databases, including PubMed, the Cochrane Library, EMBASE, Web of Science, and Scopus, were used to find relevant articles published before July 2024. In our search strategy, we used the following keywords to identify the articles. The search keywords used were “time-restricted eating”, “time restricted eating”, “TRE”, “time-restricted feeding”, “TRF”, “time restricted diet”, “fasting”, “intermittent fasting”, “intermittent energy restriction”, “energy restriction”, “CR”, “calorie restriction” OR “intermittent caloric restriction” AND “body weight”, “body mass index”, “BMI”, “fat mass”, “total fat”, “body fat rate”, “body fat percentage”, “fat free mass”, “fat-free mass”, “lean mass”, “lean body mass”, “muscle mass”, “visceral fat”, “visceral adipose tissue”, “VAT”, “body composition”, “waist size” OR “waist circumference” (Supplementary Fig. 1). A manual search of the reference lists of the related articles was also performed.

### Inclusion and exclusion criteria

In this systematic review and meta-analysis, the inclusion criteria were as follows: (1) all studies were RCTs; (2) participants were adults aged above 18 years (irrespective of health status); (3) daily fasting time in trials should be a minimum of 12 h or more, at least 6 days per week; (4) studies should report at least one of the outcome measures (body weight, BMI, fat mass, fat-free mass or WC); (5) TRE trial compared with suitable control (no restriction diet or energy restriction diet) and (6) RCTs should be peer-reviewed, and published in indexed journals. The exclusion criteria were as follows: (1) intervention trials combined with exercise, (2) studies with other intermittent fasting patterns (periodic fasting, alternate-day fasting), (3) fasting during Ramadan, (4) trials with < 3 weeks of fasting and (5) articles with insufficient or non-English data. Article search, evaluation, and selection were performed by two independent authors (YS and YL). Additional review and crosschecks were conducted by the other review authors (WY, VRL, VC). Disagreements on the inclusion or exclusion of articles were resolved by discussing with the other authors (WL, MK).

### Data extraction

Two experienced investigators (YS and YL) extracted and tabulated the following information from each included study: (1) first author name, year and country of publication, (2) participant characteristics (age, sex and number in each trial), (3) participants’ health status, (4) intervention details (fasting:eating time and duration), (5) details of outcome measures (body weight, BMI, WC, fat mass, fat mass percentage (FM%), and fat-free mass), and (6) methodologies or tools used to assess the outcome measures. Based on availability, means of pre- and postintervention or mean change data were extracted for outcome measures. The extracted data were verified by another review author (YW). Any disagreements during the data extraction were resolved by discussion with the corresponding author (MK). The other two authors (VRL and VC) were involved in the in-depth analysis and interpretation of the results. If any study reported two experimental trials and one control trial, the number of participants in control was split into half, and used as the sample size to avoid the classical mistake known as ‘double counting’ in meta-analysis (Table 1).

**Table 1:** Characteristics of the included randomized controlled trials

Study Year	Country	Participants’ Health status	Groups/Participants (n)	Sex (F/M)	Age	TRE (Fast:Eat, h)	Weight (kg)	BMI (kg/m2)	Waist (cm)	FM (kg)	FFM (kg)	Duration (wk)
Kunduraci et al. 2020 [43]	Türkiye	Adults with MS	TRE + ER:32 CON + ER:33	16/16 18/15	47.4 ± 12.3 48.7 ± 12.2	16:8	97.53 ± 15.9 88.43 ± 11.5	36.58 ± 5.26 32.82 ± 4.14	ND ND	38.79 ± 10.2 32.89 ± 8.96	58.73 ± 10.4 55.54 ± 9.13	12 wk
Cienfuegos et al. 2020 [23]	USA	Obese	4hTRE:16 6hTRE:19 CON:14	14/2 18/1 12/2	49 ± 8 46 ± 13.08 45 ± 7.48	20:4 18:6	101 ± 20 99 ± 21.79 93 ± 18.71	36 ± 4 37 ± 4.36 36 ± 3.74	ND ND ND	48 ± 12 48 ± 13.08 43 ± 11.22	52 ± 8 50 ± 13.08 48 ± 11.22	8 wk
Thomas et al. 2020 [45]	USA	Obese	eTRE + ER:41 CON + ER:40	34/7 35/5	38.3 ± 7.9 37.8 ± 7.8	14:10	96.1 ± 18.1 93.4 ± 18.4	34.6 ± 5.8 33.7 ± 5.6	ND ND	41.2 ± 11.2 40.1 ± 11.9	53.7 ± 9.8 52.1 ± 9.1	12 wk
Lowe et al. 2020 [44]	USA	Obese	TRE:25 CON:25	12/13 10/15	43.3 ± 11.8 44.4 ± 10.7	16:8	92.6 ± 15.2 93.0 ± 13.3	31.5 ± 4.5 31.3 ± 3.5	ND ND	30.3 ± 7.27 30.7 ± 7.27	60.0 ± 10.66 59.7 ± 10.66	12 wk
Chow et al. 2020 [50]	USA	Overweight or obese	TRE:11 CON:9	9/2 8/1	46.5 ± 12.4 44.2 ± 12.3	16:8	95.2 ± 22.6 100.9 ± 28.1	33.8 ± 7.6 34.4 ± 7.8	ND ND	41.1 ± 16.8 45.6 ± 20.7	50.0 ± 9.8 51.1 ± 8.7	12 wk
Phillips et al. 2021 [54]	Switzerland	Adults with one MS component	TRE:25 CON:20	ND ND	44.3 ± 12.8 42.5 ± 14.0	12:12	79.6 ± 15.9 77.5 ± 13.8	28.0 ± 4.1 27.0 ± 4.0	92.4 ± 11.6 90.1 ± 11.0	ND ND	ND ND	26 wk
Pureza et al. 2021 [39]	Brazil	Obese females	TRE + ER:31 CON + ER:27	31/0 27/0	31.80 ± 6.96 31.03 ± 7.16	12:12	81.25 ± 13.5 80.25 ± 9.4	33.53 ± 4.53 33.12 ± 3.63	102.8 ± 10 98.8 ± 9.6	ND ND	ND ND	52 wk
Che et al. 2021 [49]	China	T2D	TRE:60 CON:60	29/31 26/34	48.21 ± 9.32 48.78 ± 9.56	14:10	75.06 ± 4.42 74.68 ± 4.35	26.42 ± 1.96 26.08 ± 2.14	ND ND	ND ND	ND ND	12 wk
Mayra et al. 2022 [48]	USA	Healthy	eTRE:8 CON:10	0/8 1/9	25.1 ± 4.1 21.8 ± 3.8	18:6	59.5 ± 6.4 67.6 ± 10.6	22.3 ± 2.2 24.4 ± 2.4	69.5 ± 4.8 75.7 ± 7.0	ND ND	ND ND	4 wk
Xie et al. 2022 [46]	China	Healthy	eTRE:28 mTRE:26 CON: 28	24/4 19/7 21/7	28.68 ± 9.71 31.08 ± 8.44 33.57 ± 11.6	16:8 16:8	61.1 ± 8.8 61.0 ± 11.7 61.2 ± 9.9	22.7 ± 3.1 21.4 ± 2.2 21.5 ± 2.9	ND ND ND	ND ND ND	ND ND ND	5 wk
Haganes et al. 2022 [21]	Norway	Obese females	TRE:33 CON:33	33/0 33/0	36.2 ± 5.9 36.4 ± 6.2	14:10	91.0 ± 10.8 95.0 ± 11.2	31.8 ± 3.3 33.1 ± 4.2	ND ND	37.3 ± 7.6 39.5 ± 10.1	30.0 ± 2.9 31.0 ± 3.1	7 wk
Jamshed et al. 2022 [42]	USA	Obese	eTRE:45 CON:45	35/10 37/8	43 ± 10 43 ± 11	16:8	112.3 ± 20.1 105.3 ± 20.7	40.1 ± 6.6 39.2 ± 6.8	ND ND	54.4 ± 13.9 50.6 ± 14.8	57.8 ± 11.4 54.8 ± 10.7	14 wk
Liu et al. 2022 [12]	China	Obese	TRE + ER:69 CON + ER:70	33/36 35/35	31.6 ± 9.3 32.2 ± 8.8	16:8	88.4 ± 10.2 87.9 ± 12.8	31.8 ± 2.9 31.3 ± 2.6	99.4 ± 7.8 99.2 ± 9.1	33.0 ± 7.3 33.2 ± 6.3	51.2 ± 7.8 50.9 ± 9.1	52 wk
Queiroz et al. 2022 [24]	Brazil	Overweight or obese	eTRE + ER:13 dTRE + ER:11 CON + ER:13	11/2 9/2 11/2	33 ± 6 30 ± 7 26 ± 4	16:8 16:8	83.4 ± 10.6 83.9 ± 15.6 81.4 ± 13.1	30.8 ± 3 30.5 ± 3 30.1 ± 3	ND ND ND	35.9 ± 6.62 36.6 ± 7.44 36.2 ± 5.46	45.0 ± 5.87 45.0 ± 6.10 43.0 ± 8.11	8 wk
Wei et al. 2023 [47]	China	Obese and NAFLD	TRE + ER:45 CON + ER:43	21/24 18/25	32.3 ± 10.5 31.7 ± 8.3	16:8	88.9 ± 10.9 91.5 ± 13.6	32.2 ± 3.4 32.2 ± 3.2	100.4 ± 8.2 102.3 ± 9.5	33.8 ± 7.4 35.0 ± 7.2	51.4 ± 7.9 52.7 ± 8.8	52 wk
Lin et al. 2023 [51]	USA	Obese	TRE:30 CON:30	25/5 25/5	44 ± 12 44 ± 13	16:8	100 ± 17 102 ± 17	37 ± 6 38 ± 5	109 ± 13 110 ± 13	46 ± 11 47 ± 10	50 ± 10 51 ± 8	52 wk
Liu et al. 2023 [40]	China	Healthy females	TRE:19 CON:19	19/0 19/0	20.29 ± 1.79 20.08 ± 1.76	16:8	56.34 ± 4.70 54.14 ± 5.81	21.63 ± 1.24 20.32 ± 1.06	ND ND	21.6 ± 30.99 20.3 ± 38.25	34.7 ± 26.18 33.8 ± 28.01	8 wk
Pavlou et al. 2023 [53]	USA	T2D	TRE:25 CON:25	18/7 18/7	56 ± 13 54 ± 11	16:8	105 ± 25 107 ± 22	39 ± 9 39 ± 7	117 ± 13 121 ± 15	43 ± 10 47 ± 14	54 ± 13 53 ± 10	26 wk
Güner et al. 2024 [52]	Türkiye	Healthy	TRE:15 CON:15	11/4 11/4	28.93 ± 4.30 27.13 ± 2.36	16:8	61.8 ± 19.26 63.8 ± 15.93	23.06 ± 3.37 23.83 ± 3.41	ND ND	19.01 ± 4.79 17.73 ± 4.79	25.43 ± 6.80 25.23 ± 5.57	4 wk
Irani et al. 2024 [41]	Iran	Overweight/obese females	TRE + ER:29 CON + ER:27	29/0 27/0	43.56 ± 9.26 40.97 ± 8.33	14:10	80.43 ± 8.94 83.46 ± 10.2	30.78 ± 3.22 31.80 ± 3.76	90.01 ± 8.1 94.70 ± 9.0	30.95 ± 5.13 32.73 ± 5.46	44.49 ± 3.75 45.88 ± 5.02	8 wk

*F* female, *M* male, *TRE* time-restricted eating, *BMI* body mass index, *FM* fat mass, *FFM* fat-free mass, *wk* week, *eTRE* early time-restricted eating, *mTRE* midday time-restricted eating, *dTRE* delayed time-restricted eating, *CON* control group, *ER* energy restriction, *ND* no data, *MS* metabolic syndrome, *T2D* type 2 diabetes, *NAFLD* nonalcoholic fatty liver disease

### Study outcomes and assessment methodologies

The outcome measures of this study are anthropometrics (body weight, BMI, and WC) and body composition variables (fat mass, FM percentage, and fat-free mass). The tools and methodologies, including bioelectrical impedance analysis (BIA), dual-energy X-ray absorptiometry (DEXA), or any other digital apparatus that used to measure the specific variables, were recorded.

### Quality assessment

According to the Cochrane risk of bias (RoB) assessment tool version 2 (RoB2) [37], two review authors independently evaluated the risk of bias of all included studies. This updated RoB2 guidelines contain five different bias domains, including bias arising from ‘randomization process’, ‘deviations from intended interventions’, ‘missing outcome data’, ‘measurement of the outcome’, and ‘selection of the reported results’. The quality of each bias was rated as “low risk of bias (+)”, “high risk of bias (−)”, or “some concerns (?)”, and labeled them with green, red, and yellow, respectively. Differences in quality assessment were resolved by discussion with the other two review authors.

### Data analyses and statistical methods

All the statistical analyses were performed using the Review Manager Software (RevMan5.4.1). We used the mean difference and confidence intervals (CIs) to assess the effect of the interventions on the outcome measures. For the meta-analysis, we used the mean differences between pre- and postintervention measurements in both the experimental and control trials. If any study did not report the mean differences, we extracted pre- (baseline) and postintervention (after intervention) mean and standard deviation (SD) values, and calculated the mean differences using the following formula [38].

$$\mathrm{SD}\;\mathrm{change}=\sqrt{\left[\mathrm{SDpre}\right]2+\left[\mathrm{SDpost}\right]2-2\times\mathrm{corr}\times\mathrm{SDpre}\times\mathrm{SDpost}}$$

The I^2^ test was used to determine the heterogeneity of each trial, and 25%, 50%, and 75% of the values represented low, medium, and high statistical heterogeneity, respectively. If heterogeneity was statistically significant (p < 0.05), we used a random effect model for the analysis. If heterogeneity was not statistically significant (p > 0.05), the fixed effect model was used for the analysis. The included trials were categorized into two subgroups. The data in each subgroup, that is, time-restricted eating (TRE) and TRE plus energy restriction (TRE + ER), were compared with those from no restriction (NR) trials and ER trials, respectively.

## Results

### Systematic search findings

**Fig. 1:**
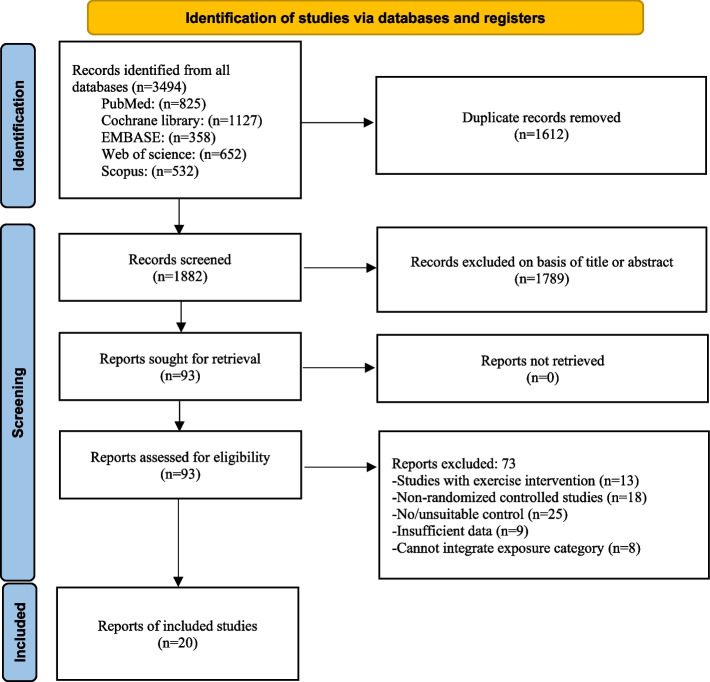
Schematic representation of the article search strategy according to PRISMA

We retrieved a total of 4006 articles from the databases, 825 from PubMed, 1127 from the Cochrane Library, 358 from EMBASE, 652 from Web of Science, and 532 from Scopus. Initially, 1612 duplicate records were removed, and the remaining 1882 articles were screened for relevance. After screening the titles and abstracts, 1789 records were excluded for various reasons, including animal studies, reviews, letters, conference abstracts, and non-English articles. Then, the full texts of 93 records were obtained and assessed for eligibility. After reading the full text of each article, 73 records were excluded for the following reasons: studies involving exercise intervention (n = 13), non-RCTs (n = 18), no/unsuitable controls (n = 25), insufficient data for analysis (n = 9), and no data of anthropometric or body composition measures (n = 8). Finally, 20 articles were included in this systematic review and meta-analysis. The detailed number of articles in each step is clearly depicted in Fig. 1.

### Characteristics of the included studies

A total of 20 studies comprising 1242 participants were included in this analysis. All included studies were RCTs, intercontinental, and were conducted in the USA (n = 8), China (n = 5), Brazil (n = 2), Türkiye (n = 2), Iran (n = 1), Norway (n = 1), and Switzerland (n = 1) (Table 1). The age of the participants ranged from 21 to 49 years. Four studies recruited only female participants [21, 39–41], fifteen studies recruited both male and female participants [12, 23, 24, 42–53], and one study did not report the sex of the participants [54]. These studies intended to evaluate the effect of TRE on clinical outcomes (anthropometric or body composition) in people with different health status. Regarding health status, four studies were conducted on healthy people [40, 46, 48, 52], eleven studies were conducted on individuals with overweight or obesity [12, 21, 23, 24, 39, 41, 42, 44, 45, 50, 51], two studies were conducted on adults with metabolic syndrome [43, 54], two studies were conducted on people with type 2 diabetes [49, 53], and one study was conducted on patients with nonalcoholic fatty liver disease (NAFLD) [47] (Table 1). The outcome measures data of participants in the TRE trials were compared with those in the no restriction trial or ER trial. Of the twenty included articles, twelve studies used DEXA, seven studies used BIA, and one study used a digital scale to assess the components of anthropometrics and body composition (Supplementary Table 2).

The fasting time in TRE trials ranged from 12 to 20 h, where it was 12 h in two studies [39, 54], 14 h in four studies [21, 41, 45, 49], 16 h in fourteen studies [12, 24, 40, 42–44, 46, 47, 50–53], 18 h in two studies [23, 48], and 20 h in only one study [23]. The TRE intervention duration ranged from 4 to 52 weeks, and 16 of 20 studies were conducted for at least 8 weeks (Table 1). Next, we noticed three studies with two TRE arms and only one control arm. To be specific, Cienfuegos et al., [23] used 4 and 6 h TRE arms, Queiroz et al., [24] adopted early and delayed TRE (eTRE, dTRE) intervention, and Xie et al., [46] used early and midday TRE (eTRE, mTRE) arms (Table 1).

### Risk of bias of the included RCTs

**Fig. 2:**
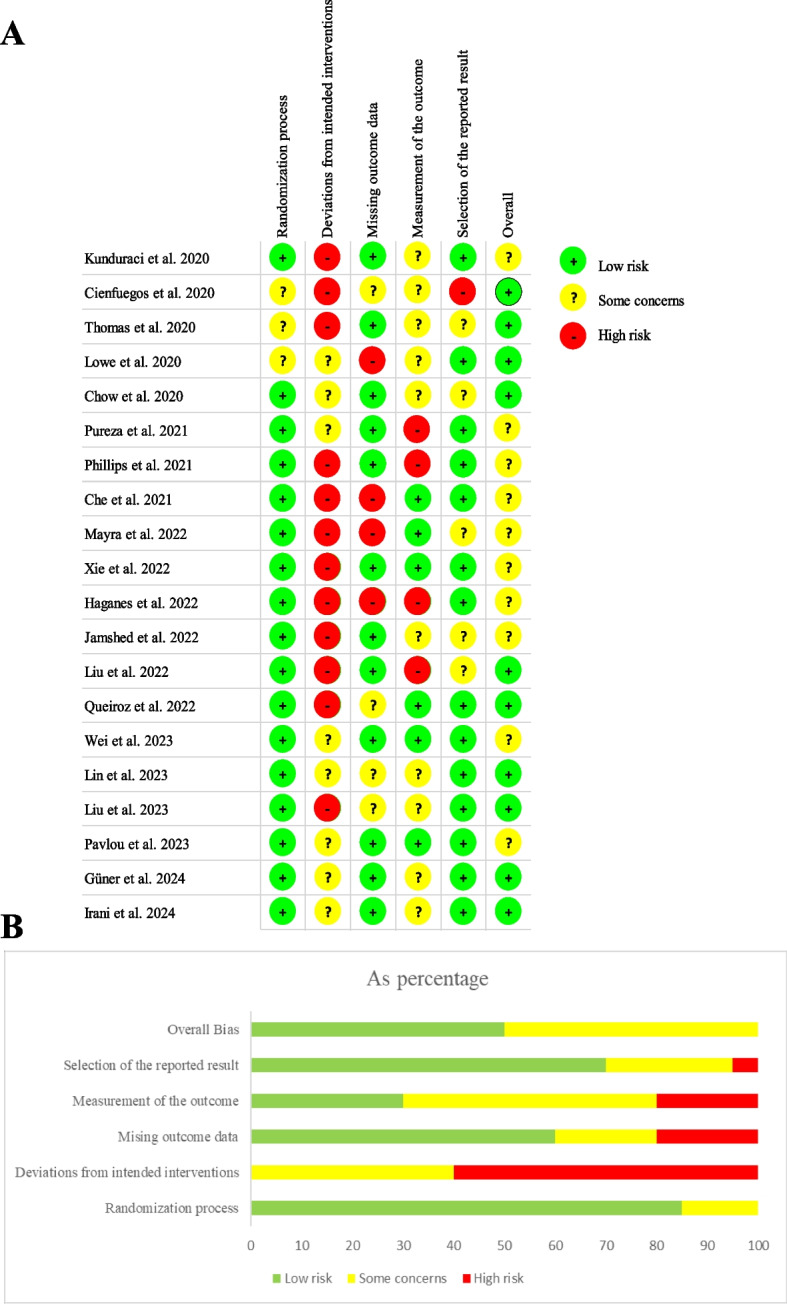
Assessment of risk of bias for all included trials (**A**) Judgement showing of risk of bias for each included article. (**B**) Breakdown of risk of bias judgement for each domain across all studies. Red (-) indicates high risk of bias; yellow (?) indicates some concerns and green (+) indicates low risk of bias

The risk of bias of the included studies assessed by RoB2, is shown in Fig. 2A and B. Of the 20 included studies, none were judged to have a high risk of bias for the ‘randomization process’; however, three studies were found to have some concerns [23, 44, 45]. Because of the nature of the experimental intervention, blinding of all participants and study personnel was not feasible. Thus, twelve studies were assessed to have a high risk of bias for the ‘deviations from intended interventions’ [12, 21, 23, 24, 40, 42, 43, 45, 46, 48, 49, 54], and eight studies showed some concerns [39, 41, 44, 47, 50–53]. Next, ‘missing outcome data’ showed some concerns in ten studies[23, 40–45, 50–52], and four studies were judged to have high risk of bias [12, 21, 39, 54]. Only four studies were assessed to have high risk of bias in ‘measurement of the outcome’ [21, 44, 48, 49]. Bias in ‘selection of the reporting results’ was judged to be low risk or no risk for several studies, some concerns in five studies [12, 42, 45, 48, 50], and high risk of bias in only one study [23]. No high risk of bias was detected for ‘overall bias’, but half of the studies showed some concerns (Fig. 2A and B).

### Meta-analysis results

#### TRE decreases body weight in adults

**Fig. 3:**
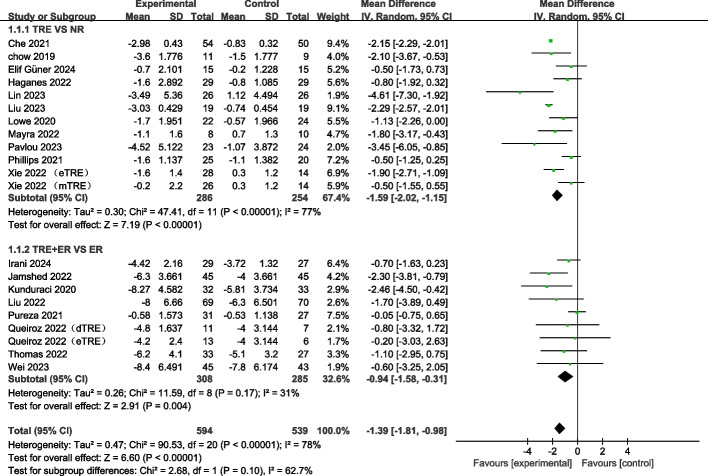
Forest plot analysis showing the effects of TRE and TRE + CR on body weight changes. TRE, time-restricted eating; NR, no restriction; CR, calorie restriction; eTRE, early time-restricted eating; mTRE, midday time-restricted eating; dTRE, delayed time-restricted eating

Nineteen RCTs [12, 21, 24, 39–54] containing 21 arms reported the changes in body weight. The pooled meta-analysis results using the random-effects model demonstrated a significant decrease in body weight following TRE (WMD: −1.39 kg, 95% CI: −1.81 to −0.98,* p* < 0.00001, I^2^ = 78%) (Fig. 3). The subgroup analysis showed that TRE independently decreased body weight (WMD: −1.59 kg, 95% CI: −2.02 to −1.15; *p* < 0.00001) compared to no restriction. Compared to ER, TRE + ER also significantly decreased body weight (WMD: −0.94 kg, 95% CI: −1.58 to −0.31, *p* = 0.004). These findings reveal that both TRE and TRE + ER interventions promote weight loss in adults, and no significant difference between the subgroups (Fig. 3).

#### TRE decreases fat mass with or without combination of ER

**Fig. 4:**
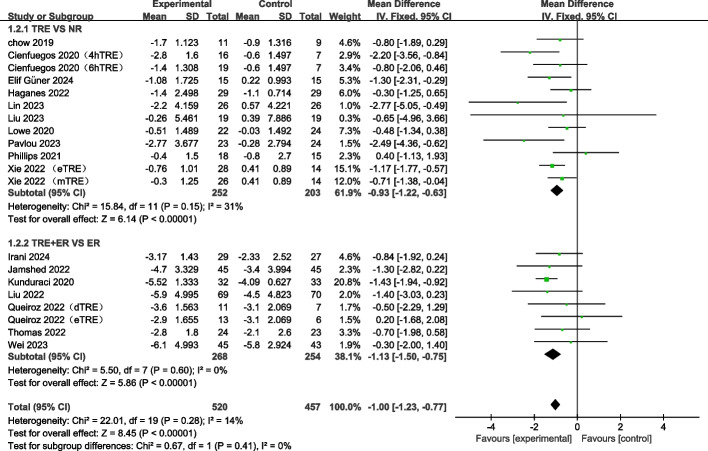
Forest plot analysis showing the effects of TRE and TRE + CR on fat mass changes. TRE, time-restricted eating; NR, no restriction; CR, calorie restriction; eTRE, early time-restricted eating; mTRE, mid-day time-restricted eating; dTRE, delay time-restricted eating; 4hTRE, 4 h time-restricted eating; 6hTRE, 6 h time-restricted eating

Seventeen studies containing 20 arms, and a total of 977 individuals (intervention = 520, control = 457) reported changes in fat mass as an outcome measure [12, 21, 23, 24, 40–47, 49, 51–54]. The meta-analysis revealed that TRE significantly decreased fat mass (WMD: −1.00 kg, 95% CI: −1.23 to −0.77, *p* < 0.00001, I^2^ = 14%) (Fig. 4). Subgroup analysis revealed that TRE alone and TRE combined with ER also caused a significant decrease in fat mass (TRE: WMD: −0.93 kg, 95% CI: −1.22 to −0.63; *p* < 0.00001; TRE + ER: WMD: −1.13 kg, 95% CI: −1.50 to −0.75; *p* < 0.00001). These findings imply that TRE intervention can decrease fat mass with or without the combination of ER (Fig. 4).

#### TRE and TRE + ER had no effect on fat mass percentage

**Fig. 5:**
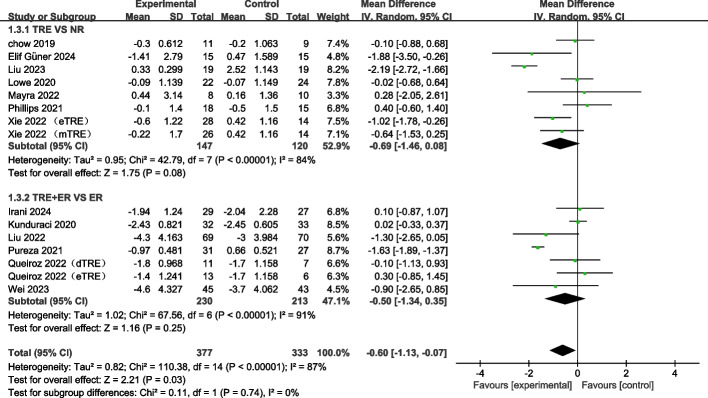
Forest plot analysis showing the effects of TRE and TRE + CR on fat mass percentage. TRE, time-restricted eating; NR, no restriction; CR, calorie restriction; eTRE, early time-restricted eating; mTRE, midday time-restricted eating; dTRE, delayed time-restricted eating

The effect of TRE with or without a combination of ER on FM% was assessed in a total of thirteen studies [12, 24, 39–41, 43, 44, 46–48, 50, 52, 54]. The overall results from the meta-analysis showed that TRE (irrespective of ER), slightly decreased FM% (WMD: −0.60%, 95% CI: −1.13 to −0.07; *p* = 0.03, I^2^ = 87%). However, subgroup analysis showed that neither TRE alone (WMD: −0.69%, 95% CI: −1.46 to 0.08; *p* = 0.08) nor TRE + ER (WMD: −0.50%, 95% CI: −1.34 to 0.35; *p* = 0.25) had a significant effect on decreasing FM% (Fig. 5).

#### TRE and TRE + ER decreases fat-free mass

**Fig. 6:**
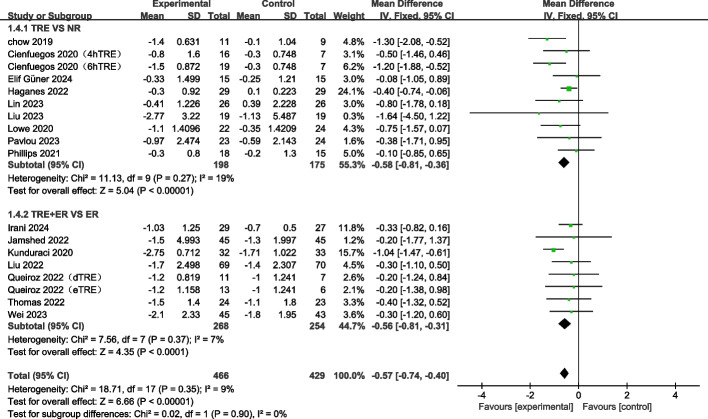
Forest plot analysis showing the effects of TRE and TRE + CR on fat-free mass changes. TRE, time-restricted eating; NR, no restriction; CR, calorie restriction; eTRE, early time-restricted eating; dTRE delay time-restricted eating; 4hTRE, 4 h time-restricted eating; 6hTRE, 6 h time-restricted eating

Fat-free mass was reported by sixteen studies comprising 895 participants [12, 21, 23, 24, 40–45, 47, 50–54]. The overall effect of TRE on fat-free mass reduction was significant (*p* < 0.00001), and the effect size was −0.57 kg, with I^2^ = 9% (Fig. 6). Next, we performed subgroup analysis, and observed a significant reduction in fat-free mass after TRE (WMD: −0.58 kg, 95% CI: −0.81 to −0.36, *p* < 0.00001) as well as after TRE combined with ER (WMD: −0.56 kg, 95% CI: −0.81 to −0.31, *p* < 0.0001) interventions (Fig. 6).

#### TRE independently and also in combination with ER decreases BMI

**Fig. 7:**
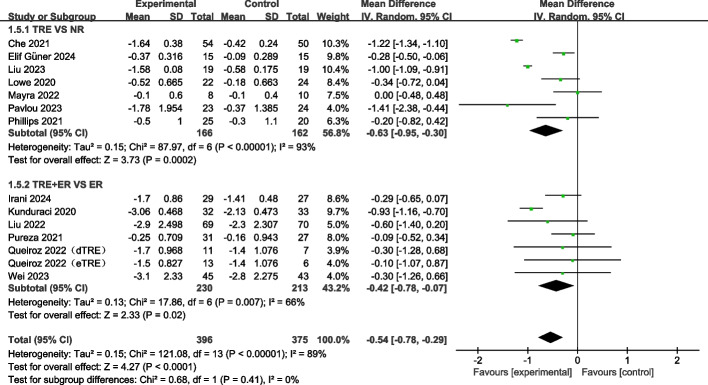
Forest plot analysis showing the effects of TRE and TRE + CR on body mass index changes. TRE, time-restricted eating; NR, no restriction; CR, calorie restriction; eTRE, early time-restricted eating; dTRE delay time-restricted eating

A total of thirteen studies (771 participants) that reported BMI values were included in our analysis [12, 24, 39–41, 43, 47–50, 52–54]. As shown in Fig. 7, the pooled outcome revealed that TRE intervention significantly decreased BMI in adults (WMD: −0.54 kg/m^2^, 95% CI: −0.78 to −0.29, *p* < 0.0001, I^2^ = 89%). The results from subgroup analysis revealed a significant decrease in BMI when participants underwent TRE intervention alone (WMD: −0.63 kg/m^2^, 95% CI: −0.95 to −0.30, *p* = 0.0002). Similarly, TRE combined with ER also resulted in a significant decrease in BMI (WMD: −0.42 kg/m^2^, 95% CI: −0.78 to −0.07, *p* = 0.02) (Fig. 7).

#### TRE combined with ER decreases waist circumference

**Fig. 8:**
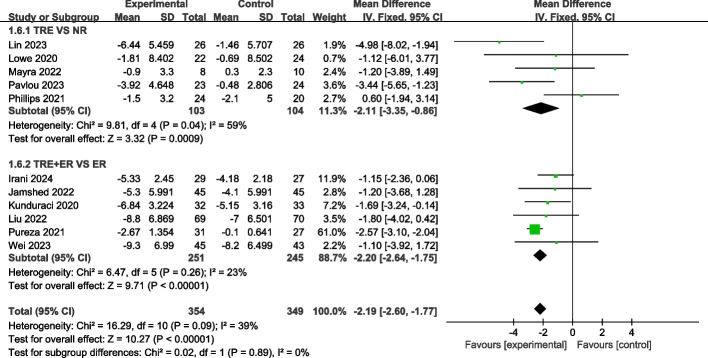
Forest plot analysis showing the effects of TRE and TRE + CR on changes in waist circumference. TRE, time-restricted eating; NR, no restriction; CR, calorie restriction

Eleven studies that reported WC data (703 participants) were analyzed to determine the effect of TRE and TRE + ER on changes in WC size [12, 39, 41–44, 47, 48, 51, 53, 54]. The pooled meta-analysis results revealed a significant decrease in WC after TRE (WMD: −2.19 cm, 95% CI: −2.60 to −1.77; *p* < 0.00001, I^2^ = 39%) (Fig. 8). From the subgroup analysis results, it is clear that TRE intervention independently decreased WC (WMD: −2.11 cm, 95% CI: −3.35 to −0.86; *p* = 0.0009). Furthermore, TRE + ER also resulted in a significant decrease in WC of 2.20 cm, and the 95% CI was −2.64 to −1.75 (*p* < 0.00001) (Fig. 8). These results indicate that TRE, independently as well as in combination with ER, is effective in decreasing the WC of adults.

## Discussion

To the best of our knowledge, this is the first systematic review and meta-analysis to explore the efficacy of TRE with or without combination of ER on anthropometric and body composition variables among adults. The included RCTs showed no high risk of bias for the “randomization process” and “overall bias”. Our meta-analysis (pooled) results demonstrated that TRE intervention significantly decreased anthropometric and body composition variables, including body weight, BMI, WC, fat mass, FM%, and fat-free mass in adults. The subgroup analysis results revealed that TRE independently (compared to no restriction), and TRE combinedly with ER (compared to ER) also substantially decreased body weight, fat mass, fat-free mass, BMI, and WC, but no differences between the subgroups. However, FM% was not significantly changed with either TRE alone or TRE + ER intervention. Our findings imply that practicing TRE alone or in combination with ER is beneficial for weight loss and fat mass reduction, but not for fat-free mass maintenance among adults.

Anthropometrics and body composition variables are the key components for maintaining overall health and longevity, and are typically influenced by several factors, including time and type of nutritional intake [3, 41, 55]. Changes in anthropometrics, particularly weight gain is associated with increased risk of obesity, type 2 diabetes, and all-cause mortality [56], while weight loss is associated with reduced diabetic complications and improved physical functioning [57, 58]. In our study, TRE practice substantially decreased body weight with or without combination of ER, indicating the importance of daily eating window for weight loss. Similar to our TRE results, a previous meta-analysis reported decreased body weight and attenuated metabolic disorders in adults with TRE [31]. In contrast, a recent meta-analysis stated that TRE with ER can decrease body weight, while TRE with non-ER had no significant effect on body weight in adults with overweight and obesity [32]. These discrepancies might be due to the differences in TRE protocols and/or participants’ characteristics. For instance, Huang et al., [32] included eight RCTs with an eating window of 8 h, and the participants were adults with overweight and obesity. Whereas, we included 20 RCTs with an eating window ranging from 4 to 12 h, and the participants are adults with normal weight, overweight, or obesity. Our findings further emphasized that the weight loss change with TRE practice was slightly higher (mean difference −1.59 kg, TRE vs NR) than that of the weight loss change with TRE + ER practice (mean difference −0.94 kg, TRE + ER vs ER). However, the magnitude of weight loss change with TRE practice appears to be moderate in our study [59]. These findings suggest that the daily eating window (total eating time) is important than the daily energy intake for effective weight loss. It is hypothesized that extended daily fasting hours during TRE could promote the mobilization of free fatty acids and increase fat oxidation, which then results in weight loss [19].

Dietary interventions (reduced energy intake) with or without physical exercise are effective strategies for managing obesity and obesity-associated diseases [60]. Not surprisingly, we found decreased fat mass with TRE alone and also in combination with ER, while FM% remained unchanged. A previous study on patients with obesity reported that both TRE and daily caloric-restricted eating reduced fat mass after 12 months, but no differences between the groups [12]. Similar to our findings, a meta-analysis reported decreased fat mass and body weight with TRE. However, they did not address the effect of TRE combined with ER on fat mass reduction, and the results were limited by the short duration of TRE intervention and small sample size [19]. In our study, the TRE duration was 8 weeks or more in most trials (16 of 20 trials), with a large number of participants (*n* = 1242). Greater restriction of the eating window without restriction of energy intake was associated with greater loss of fat mass and visceral fat in adults with overweight [50]. The greater loss of fat mass with TRE compared to a normal diet (no differences in energy intake) cannot be simply explained by changes in diet quality or quantity; instead, different temporal meal distributions might be the key factor [61]. Practicing early TRE (6 h) reported to alter the temporal patterns (increase fullness, decrease desire to eat) and increase fat oxidation in adults with overweigh, which may have contributed to fat loss [62]. Early TRE (8 h) plus ER is reported to be more effective than control eating (> 12 h) plus ER in decreasing body fat in adults with obesity [42], and this may be associated with increased fat burning during prolonged fasting hours in a day [63]. The importance of the eating window for fat mass reduction was further supported by an RCT in which consuming a single meal/day as dinner (4 h TRE, 8-week) decreased fat mass by 13% and preserved fat-free mass compared to consuming the same amount of energy as breakfast, lunch and dinner in healthy middle-aged adults [64]. However, the effect of consuming a single meal, particularly dinner, on several health-related clinical outcomes is debatable; therefore, early TRE might be advantageous.

Next, we found decreased fat-free mass with TRE and TRE + ER, which appears to be a concern regarding the intervention effect on body composition. These results differ from the previous meta-analyses, which showed unchanged fat-free mass with TRE compared to the baseline [19] as well as TRE compared to controls (no restriction) [33]. However, our findings are similar to a previous study that reported decreased fat-free mass and body weight with 8-h TRE in overweight and obese patients with chronic kidney disease [65]. The differences in meal timing, eating window, daily energy, or protein distribution during TRE may cause disparities in the maintenance of fat-free mass [66]. Notably, Stratto et al., [66] reported that an 8-h eating window with a 25% energy deficit for 4-week decreased body weight in male adults, which may be attributed to decrease in fat-free mass and fat mass. This could be due to a lack of sufficient protein intake and/or the absence of a structured exercise regimen [66]. A systematic review of overweight or obese cohorts reported that the magnitude of ER was positively associated with the percentage of weight loss as fat-free mass [67]. These findings highlight the importance of sufficient protein intake (≥ 2 g/kg/day) in adults to preserve the fat-free mass during energy-restricted interventions [68]. We assume that the decreased total energy intake, differences in protein intake, or changes in energy expenditure during TRE may contribute to decrease in fat-free mass in adults; however, this phenomenon is yet to be established. On the other hand, we found no significant change in fat mass percentage in the TRE or TRE + ER trials, which may be attributed to the loss of fat-free mass.

Another important finding in our analysis is that both TRE and TRE + ER interventions significantly reduced BMI and WC in a similar fashion to weight loss and fat loss. In contrast to our findings, a recent meta-analysis concluded that TRE alone had no effect on BMI or WC, while decreased body weight and fat mass in adults with overweight [31]. An RCT showed that a 12-week TRE intervention with 8-h eating window effectively reduced body weight but not WC or fat mass in adults with overweight and obesity [44]. Evidence from clinical trials confirmed that both TRE plus ER and ER alone can reduce WC and BMI in patients with obesity, but combining TRE with ER offers no additional advantages over ER alone [12, 42]. Similarly, a meta-analysis (4 studies) reported that both TRE plus ER and ER interventions similarly decreased WC in adults with overweight and obesity, while no additional benefits from combined intervention or varying TRE schedules [69]. The changes in WC and BMI metrics during TRE intervention are influenced by the length of the daily eating window and/or the amount of daily energy intake [27, 70]. However, the diverse results of TRE from the meta-analyses [31, 69] may stem from the variations in the TRE protocols (eating window, eating schedule, intervention duration), energy intake, participants’ age, health status, or baseline conditions. The Consensus Statement recommends that routine measurement of WC in clinical practice can provide a valid additional information for guiding patient and disease treatment [71]. This Consensus Statement further emphasized that stratifying patients according to risk cannot be achieved without the inclusion of WC measurements in clinical practice [71]. Previous studies have shown that WC provides both independent and additive predictive values beyond BMI for assessing morbidity and mortality risks [71, 72]. Furthermore, the waist-BMI ratio was independently associated with overall and cardiovascular mortality, and had a better predictive ability than other conventional anthropometric measures for obesity [73]. Since both BMI and WC are intrinsically involved in the progression of obesity, practicing TRE with or without ER may be an effective strategy to lower these metrics, particularly in adults with overweight or obesity. This approach could help mitigate the obesity and obesity-associated diseases.

### Limitations

Although TRE demonstrated significant effects on anthropometrics and body composition, some limitations should be considered. Most participants included in our analyses were healthy patients with overweight and obesity; therefore, our findings may not be applicable to patients with other diseases, like cancer or cardiovascular diseases. Given the importance of preserving fat-free mass, the changes in fat-free mass observed in our study (TRE and TRE + ER groups) should be further validated. Due to lack of adequate numbers of RCTs that reported energy intake data, we are unable to claim that the beneficial effects of TRE are mainly due to the reduced energy intake. In addition, our study did not address other potential confounders, like age, gender, sample size, and variations in body composition assessment tools, which could influence the intervention effect.

## Conclusions

For the first time, our meta-analyses demonstrated that TRE intervention improved anthropometric and body composition variables, including body weight, fat mass, BMI, and WC, but not fat-free mass in adults. Subgroup analyses showed that TRE independently promoted weight loss by decreasing body weight, fat mass, and BMI when compared to NR. TRE combined with ER also showed similar beneficial effects on anthropometric and body composition variables when compared to ER. However, TRE intervention alone and also in combination with ER lead to a similar decrease of fat-free mass. Our findings suggest that combining TRE with ER has no additional beneficial effects on weight loss in adults.

## Supplementary Information

Supplementary Material 1. PRISMA checklist.

Supplementary Material 2. Figure S1. Search strategy performed on PubMed. Table S2. Anthropometric or body composition variables and assessment methodologies in RCTs.

## Data Availability

No datasets were generated or analysed during the current study.

## Abbreviations

4hTRE: 4 H time-restricted eating
6hTRE: 6 H time-restricted eating
BIA: Bioelectrical impedance analysis
BMI: Body mass index
CI: Confidence intervals
CR: Calorie restriction
dTRE: Delayed time-restricted eating
DEXA: Dual-energy X-ray absorptiometry
eTRE: Early time-restricted eating
ER: Energy restriction
FFM: Fat-free mass
FM: Fat mass
FFM%: Fat mass percentage
NAFLD: Nonalcoholic fatty liver disease
NR: No restriction
PRISMA: Preferred reporting items for systematic reviews and meta-analyses
PROSPERO: Prospective register of systematic reviews
RCT: Randomized controlled trial
RoB2: Risk of bias version 2
SD: Standard deviation
TRE: Time-restricted eating
VAT: Visceral adipose tissue
WC: Waist circumference
WHO: World Health Organization
WMD: Weighted mean difference
