# Supplementary material for: Efficiency of time-restricted eating and energy restriction on anthropometrics and body composition in adults: a systematic review and meta-analysis of randomized controlled trials
Source: Int J Behav Nutr Phys Act. 2025 Sep 29;22:121. doi: 10.1186/s12966-025-01812-w (PMC12487415; doi:10.1186/s12966-025-01812-w)
Supplement: Supplementary file 2 — Supplementary Material 2. Figure S1. Search strategy performed on PubMed. Table S2. Anthropometric or body composition variables and assessment methodologies in RCTs. [file 12966_2025_1812_MOESM2_ESM.docx]

Efficiency of Time-Restricted Eating and Energy Restriction on Anthropometrics and Body Composition in Adults: A Systematic Review and Meta-Analysis of Randomized Controlled Trials

Yichao Sun^1†^, Yubo Liu^1†^, Weibing Ye^1^, Veeranjaneya Reddy Lebaka^2^, Venkatrayulu Chenji^3^, Weiping Li^4^ and Mallikarjuna Korivi^1^*

**Supplementary data**

**Supplementary Figure 1.** Search strategy performed on PubMed.

(("randomized controlled trial"[Title/Abstract] OR "randomized"[Title/Abstract] OR "placebo"[Title/Abstract] OR "Clinical Trials"[Title/Abstract] OR "Randomized Trials"[Title/Abstract] OR "Randomized Clinical"[Title/Abstract] OR "Controlled Clinical Trials"[Title/Abstract] OR "Randomized"[Title/Abstract] OR "random"[Title/Abstract] OR "sham"[Title/Abstract] OR "blind"[Title/Abstract] OR "RCT"[Title/Abstract] OR OR[Title/Abstract]) AND ("Body Weight"[Title/Abstract] OR "Weight, Body"[Title/Abstract] OR "body weight"[Title/Abstract] OR "body mass"[Title/Abstract] OR "weight"[Title/Abstract] OR "weight loss"[Title/Abstract] OR "weight gain"[Title/Abstract] OR "FM"[Title/Abstract] OR "body fat mass"[Title/Abstract] OR "Fat mass"[Title/Abstract] OR "total fat"[Title/Abstract] OR "Lipid"[Title/Abstract] OR "Fett"[Title/Abstract] OR "body fat rate"[Title/Abstract] OR "body fat percentage"[Title/Abstract] OR "fat free mass"[Title/Abstract] OR "lean mass"[Title/Abstract] OR "Lean body"[Title/Abstract] OR "Muscle "[Title/Abstract] OR "visceral fat"[Title/Abstract] OR "visceral adipose tissue"[Title/Abstract] OR "VAT"[Title/Abstract] OR "Body Composition"[Title/Abstract] OR "Body Compositions"[Title/Abstract] OR "Composition, Body"[Title/Abstract])) AND ("time restricted feeding"[Title/Abstract] OR "time restricted eating"[Title/Abstract] OR "time restricted fasting"[Title/Abstract] OR "time restricted diet"[Title/Abstract] OR "Intermittent Fasting"[Title/Abstract] OR "Fasting, Intermittent"[Title/Abstract] OR "Feeding, Time Restricted"[Title/Abstract] OR "Time Restricted Feedings"[Title/Abstract] OR "intermittent energy restriction "[Title/Abstract] OR "intermittent caloric restriction "[Title/Abstract] OR "intermittent fasting"[Title/Abstract] OR "time-restricted feeding"[Title/Abstract] OR “calorie restriction"[Title/Abstract] OR “energy restriction"[Title/Abstract] OR “caloric restriction"[Title/Abstract] OR “CR"[Title/Abstract]).

**Supplementary Table 2.** Anthropometric or body composition variables and assessment methodologies in RCTs

| **Study details** | **Number of participants** | **Body composition variable** | **Assessed tool or method** |
| --- | --- | --- | --- |
| Kunduraci et al. 2020 | TRE+ER:32  CON+ER:33 | Weight, FM, FM%, FFM, BMI, Waist | Bioelectrical Impedance Analysis  (TANITA SC-330) (measuring tape) |
| Cienfuegos et al. 2020 | 4hTRE:16  6hTRE:19  CON:14 | Weight, FM, FFM, BMI | Dual X-ray Absorptiometry  (iDXA, General Electric) |
| Thomas et al. 2020 | eTRE+ER:41  CON+ER:40 | FM, FFM | Dual X-ray Absorptiometry  (Hologic Discovery W, Bedford, Massachusetts) |
| Lowe et al. 2020 | TRE:25  CON:25 | Weight, FM, FM%, FFM, BMI, Waist | Dual X-ray Absorptiometry |
| Chow et al. 2020 | TRE:11  CON:9 | Weight, FM, FM%, FFM, BMI | Dual X-ray Absorptiometry |
| Phillips et al. 2021 | TRE:25  CON:20 | Weight, FM%, BMI, Waist | Dual X-ray Absorptiometry |
| Pureza et al. 2021 | TRE+ER:31  CON+ER:27 | Weight, FM, BMI, Waist | Bioelectrical Impedance Analysis  (Sanny v. 1.2.2) (inelastic tape) |
| Che et al. 2021 | TRE:60  CON:60 | Weight, BMI | Digital Scale  (Shanghai Yaohua, XK3190-A12+E)  (Shanghai Keda, TZG) |
| Mayra et al. 2022 | eTRE:8  CON:10 | Weight, FM%, BMI, Waist | Bioelectrical Impedance Analysis  (Cat. No. TBF-300, Tanita, Arlington Heights, IL) (research-grade ergonomic measuring tape) |
| Xie et al. 2022 | eTRE:28  mTRE:26  CON: 28 | Weight, FM, FM%, BMI | Bioelectrical Impedance Analysis  (Omron Healthcare Co., Kyoto, Japan) |
| Haganes et al. 2022 | TRE:33  CON:33 | Weight, FM, FFM | Bioelectrical Impedance Analysis  (InBody720, Biospace CO, Korea) |
| Jamshed et al. 2022 | eTRE:45  CON:45 | Weight, FM, FFM, Waist | Dual X-ray Absorptiometry  (DEXA [iDXA; GE-Lunar Radiation Corporation]) |
| Liu et al. 2022 | TRE+ER:69  CON+ER:70 | Weight, FM, FM%, FFM, BMI, Waist | Dual X-ray Absorptiometry  (Lunar iDXA, GE Healthcare) |
| Queiroz et al. 2022 | eTRE+ER:13  dTRE+ER:11  CON+ER:13 | Weight, FM, FM%, FFM, BMI | Dual X-ray Absorptiometry  (Lunar Prodigy Primo, GE Healthcare) |
| Wei et al. 2023 | TRE+ER:45  CON+ER:43 | Weight, FM, FM%, FFM, BMI, Waist | Dual X-ray Absorptiometry  (Lunar iDXA; GE Healthcare) |
| Lin et al.2023 | TRE:30  CON:30 | Weight, FM, FFM, Waist | Dual X-ray Absorptiometry  (iDXA, GE)(measuring tape) |
| Liu et al.2023 | TRE:19  CON:19 | Weight, FM, FM%, FFM, BMI | Dual X-ray Absorptiometry  (Hologic, Horizon-Wi, United States) |
| Güner et al. 2024 | TRE:25  CON:25 | Weight, FM, FM%, FFM, BMI | Bioelectrical Impedance Analysis  (Bioelectrical Impedance Analysis, Inbody 270) |
| Irani et al.2024 | TRE:15  CON:15 | Weight, FM, FM%, FFM, BMI, Waist | Bioelectrical Impedance Analysis  (X-contact 356; Jawson Medical Co, Seoul, South Korea) (ﬂexible tape) |
| Pavlou et al.2024 | TRE+ER:29  CON+ER:27 | Weight, FM, FFM, BMI, Waist | Dual X-ray Absorptiometry |

Abbreviations: F, female; M, male; TRE, time-restricted eating; BMI, body mass index; FM, fat mass; FFM, fat-free mass; wk, week; eTRE, early time-restricted eating; mTRE, midday time-restricted eating; dTRE, delayed time-restricted eating; CON, control group; ER, energy restriction; ND, no data; MS, metabolic syndrome; T2D, type 2 diabetes; NAFLD, nonalcoholic fatty liver disease.
